# Challenging Role of Dietary Aflatoxin B1 Exposure and Hepatitis B Infection on Risk of Hepatocellular Carcinoma

**DOI:** 10.3889/oamjms.2015.032

**Published:** 2015-03-25

**Authors:** Basak Kucukcakan, Zehra Hayrulai-Musliu

**Affiliations:** *Ss. Cyril and Methodius University of Skopje, Faculty of Veterinary Medicine, Department of Food Safety, Skopje, Republic of Macedonia*

**Keywords:** Aflatoxin B1, Hepatitis B, Hepatocellular Carcinoma, Biomarker

## Abstract

Aflatoxins (AFT) are poisonous substances which are classified in Group 1 carcinogenic agents to humans by International Agency for Research on Cancer (IARC). AFT can occur naturally in food commodities (maize, corn, rice) as a result of fungal contamination in hot and humid environments. In the food, toxin contamination can remain during manufacturing and long after fungi have stopped being biologically active. Aflatoxin B1 (AFB1) is the most dominant and potent agent from all AFT. In developing countries, high exposure to AFB1 can cause chronic toxicity and usually increases the incidence of Hepatocellular Carcinoma (HCC). However, in these regions hepatitis B is the most common risk factor for HCC cases. Many researches were aimed to enlighten the mechanism and the role of two etiological agents on risk of HCC, but the obtained data was conflicting with each other. It was uncertain that the indicators/biomarkers might be the contribution of the carcinogenic status of the patient; and, the biomarker samples from the subject may only reflect the recent effects of the toxin exposure after consumption of AFB1 contaminated commodities. The studies were facing with the errors of methods which were un-fit to enlighten the possible interaction between Hepatitis B and AFB1 on contribution to HCC. It was pivotal to understand the effect of each risk factor in order to prevent and improve public health in poor and undeveloped regions. Although some of the studies evaluate AFB1 alone as a considerable factor on HCC risk, according to this review it was concluded vice versa. This study was aimed to clarify the main etiological agent of HCC where AFB1 and HBV are endangering public health. In additionally, the purpose was to enlighten the possible synergistic effect between these two factors among HCC pathogenesis. Hence forth, appropriate and right applications could be conducted in undeveloped countries in order to protect public health.

## Introduction

Hepatocellular carcinoma (HCC) is one of the direst diseases which cause mortalities more than 600,000 people each year. Chronic hepatitis B infection and aflatoxin (AFB1) exposure play crucial role in occurrence of HCC in developing countries. 4.6–28.2% of all global HCC cases may be attributable to AFB1 exposure. Moreover, if the individuals are exposed chronic hepatitis B virus (HBV) and AFB1 together, cancer risk is becoming more solemn through increasing the risk 30 times greater [1]. Hepatitis B is the most serious and frequent illness in developing countries and mainly is responsible for 80% of HCC cases around the world. Approximately, more than one million deaths occurred due to hepatitis B infection in countries, such as Africa and Asia. In these poor and undeveloped regions, people are suffering under harsh living conditions (such as poverty, scarcity and lack of sufficient health facilities and drugs) which promote HBV prevalence and AFB1 exposure. These two potent agents are endangering the public health since for a long time [2, 3].

Epoxidation of aflatoxin to 8, 9-epoxide by CP450 enzyme plays pivotal role in the pathway of the hepatocellular carcinoma; bind the guanine bases particularly on the third base of codon 249 of the p53 gene to form aflatoxin-N7-guanine (AFB1-N7-Gua). It was observed that aflatoxin can impact on p53 suppressor gene which is responsible for preventing cell cycle progression. Also, it was reported that this type of mutations dominate in the regions with high aflatoxin contamination [4, 5]. Several studies suggested that, in countries with high incidence of HCC, chronic infection with HBV was associated with high aflatoxin exposure; and within the regions of incident, infection and toxin consumption were two major etiological risk factors for HCC and further health issues, pending to AFB1 [6].

Interaction between AFB1 and HBV causing HCC was depicted by several possible mechanisms. Initially, HBV could induce the conversion AFB1 to AFB1-8, 9-epoxide by specific enzyme CP450s in both direct and indirect way. HBV originated chronic hepatitis or presence of virus itself might promote sensitization of hepatocytes during AFB1 toxicity. The metabolite has destructive impacts on DNA, by binding proteins acute toxicity occurs and viral DNA can integrate into the host genome more easily. Direct effect of AFB1 and chronic viral hepatitis both may provoke these formations [7]. Common occurrence of HBV infection and albumin serum adducts during aflatoxicosis was demonstrated in Kenya and Gambia [8, 9]. Yet, the prevalence of the codon 249 of p53 gene mutations was found significantly higher among HCC patients in South Western Nigeria. In the study, all these mutations were demonstrated among all the patients with HCC who had the HBsAg positivity [10]. In additionally, in the regions where both heavy AFB1 exposure and hepatitis B infection were common, gene p53 codon 249 mutations were observed frequently [10, 11].

Synergism between AFB1 and hepatitis B was observed through clinical studies on HBV-transgenic mice. There existed a consistent mechanism, disclosing the interaction of HBV infection which altered the expression of aflatoxin metabolism enzymes and consequent extent to DNA to which aflatoxins bind. Comprehension of the interaction mechanism, were proportional with the public health preventive measures against procurement of the HCC incidence. As it was emphasized through the course of the paper, HBV grants a lethal risk factor for HCC; and, there is an alarming necessity to understand mutagenic mechanism of HCC, sourced from the perpetrating items, threatening public health. Thus, it should be examined whether the 249 (ser) mutations occurs only in the case of exposure to both HBV and AFB1. Yet, the studies from North America, Europe and Japan delineated that chronic HBV infection alone was insufficient to commence the 249 mutation of codon p53 gene. Endemic areas made the confirmation further difficult because it was unable to clarify the distinct case of factors which were playing a role on mutation of gene due to the high prevalence of HBV infection in aflatoxin [12]. Kick et al. (2005) found out that HBV was not solely affective to cause 249 (ser) p53 mutations in plasma DNA which was related with HCC [13]. In the work of Soini et al. (1996); it was mentioned that, hepatitis B virus may activate proto-oncogenes or inactivate tumour suppressor genes, and shall commence neoplastic transformation. Also the virus shows ability to integrate into the host DNA, and bind to p53 protein; and be further hypothesised to propagate vulnerability to hepatocytes to carcinogenic damage by AFB1. In the study, it was indicated that Mesoamerica happened to be a hotspot for 249(ser) mutations in HCC, associating evidence of AFB1 contamination in the dietary scale. It was emphasized that AFB1 and hepatitis viruses were the etiological agents of HCC in the region. Also the data from different studies showed a positive correlation between dietary intake of toxin and codon 249 mutations [4].

## The role of dietary AFB1 exposure and HBV infection on codon 249 of the p53 gene mutations and HCC

Molecular epidemiological studies were depicted that a G to T mis-sense mutation at the third base of codon 249 of the p53 gene, affecting an arginine to serine substitution, occurred in high frequency (up to 67%) in human liver tumours in the regions with high risk of aflatoxin exposure. In contrast to findings, another experiments were performed, using liver tissue from liver-cancer patients in Taiwan and Japan, and were scrutinized for the presence of aflatoxin-DNA adducts (ADA) as a marker for aflatoxin exposure and an AGG to AGT transversion at codon 249 of the p53 gene. Ten per cent of samples containing ADA, indicating definite exposure of the subjects to aflatoxin, were found to harbour the codon 249 mutation, whereas 18% of the samples with no detectable adducts also contained the mutation. Since the presence of ADA in the liver tissue samples were indications of definite recent expo exposure [11].

Stern et al. (2001) observed the occurrence of HBV infection, modifying the effect of aflatoxin exposure on the proportion of tumours with any mutation in the p53 gene (i.e., aflatoxin by HBV interaction). In the conclusion, it was deduced that the presence or absence of HBV infection did not change the effect of aflatoxin exposure on the prevalence of liver tumours with p53 mutations. Moreover, it was underlined that there was no significant effect of HBV on the proportion of liver tumours with p53 mutations [14].

Apart from the case of mutations; dietary exposure to aflatoxins and determination of aflatoxin biomarkers in urine and serum, courses the fundamental method to estimate any existing correlation between toxin and HCC risk factor. For example, in Kenya - Muranga district the subjects with higher toxin intakes were residents in areas with an elevated incidence of HCC [15]. In another research, the conclusion was dictated the significant correlations between calculated ingested daily dose of AFB1 and adult male incidence of HCC in different parts of South-eastern Africa [16].

In ecological studies; AFB1 exposure was monitored through samples of urinary excretion of AFB1 metabolites and released DNA adducts. In the study of mention, data from the cross-sectional survey was not sufficient to demonstrate any relation between aflatoxin exposure and primary liver cancer (PLC) and also failed to explain the influence of the aflatoxin urinary biomarkers in the distributed analysis. Strong relation with significance was detected between HBsAg positivity and PLC occurrence. In the survey; extraordinary PLC risk was observed for HBsAg positive carriers. In carrier-positive population, additional risk was detected chiefly by nutritional and dietary practices, enhancing liver cell proliferation, as in diets with significant amounts of protein. In contrast, aflatoxin was insignificant threat of PLC risk. It was suggested that, even though aflatoxin may act as a carcinogenic initiator, toxin’s contribution to tumour generation acts as in a meagre scale for the initiation activity, routinely exposing the liver. In this study it was proposed that HBsAg positivity was necessary, yet gain insufficient factor, thus unlikely reason for PLC [17].

In the case-control study from Kenya, authors had results through systematic sampling of maize and serum from participants. The subjects with aflatoxicosis, providing serum samples, were demonstrated higher aflatoxin B1–lysine adduct concentrations in their serum than the control subjects did; and, the aflatoxin B1–lysine adducts concentrations were measured from the serum of case patients are the highest ever reported. It was commented that the serum samples concentrations with aflatoxin B1–lysine adduct showed the lethal risk of acute aflatoxicosis. The serum samples were sufficient to analyse 72 (60%) samples for hepatitis B surface antigen. The mean age of participants with positive titres was 33 years with the rate of 58% female. Eight (44%) of 18 cases had positive titres, while only 4 (7%) of 54 controls had positive. It was found that having positive hepatitis B surface antigen titres generates a risk factor for acute hepatic failure. When the data was extracted for the course of the argument, restricted to participants with negative hepatitis B titres, it was found that the subject with aflatoxin B1–lysine adduct concentrations at or above the median for this subgroup grants a risk factor for developing aflatoxicosis [8]. A case-control study in Southern Guangxi, China; distribution of serum AFB1-lysine adducts in HCC cases and controls was demonstrated. No significant difference was found in the distribution of genotypes between the cases and controls. After examinations, levels of serum AFB1- lysine adducts were detected higher in the cases than controls; yet no statistically significant difference was found. Therefore, significant association with HCC risk wasn’t demonstrated [18].

In another case-control study from Thailand, two methodological approaches were used to estimate HCC exposure. Hepatitis B virus was claimed as the major perpetrator, excluding the effect of the intake of toxin contaminated food and AFB1-albumin adducts in serum [19].

In the chort study, roles of HBV and AFB1 in the development of liver cancer were evaluated in Shanghai, China. Additionally; 1 year-long survey of market nutritive products in Shanghai was upheld to estimate the aflatoxin exposure in the study population, quantitatively. After processing, approximately 70,000 subjects per year as a follow-up, 55 cases of hepatocellular carcinoma (HCC) were identified; monoclonal antibody column and HPLCs analysis were done on 267 control samples and 55 HCC patients. AFB1-N7-gua adducts were detected in the 18 of the urine samples from case group and 31 of the control group. Also, in the control group number of AFB1 biomarker in urine was higher than the HCC case group [20]. Concurrent study was displayed the similarity [21]. In the study of Quien et al. (1994), aflatoxin was detected in rice, peanuts, wheat-flour, soy-sauce and milk several market products; and, the products, containing peanuts and soya-sauce had the highest level of aflatoxin contamination. It was estimated that, there was no significant association between dietary AFB1 level and HCC risk among the cohort members; after adjustment for HBsAg positivity. Among all cohort subjects, in dietary AFB1 (over 113 μg/kg) exposed population (n = 24013), only 16 HCC cases were observed. However, number of observed HCC cases were higher (n = 25) among population (n = 23547) when the toxin exposure was between 71-113 μg/kg. 14 HCC cases were detected among the rest of population (n = 21833) when the AFB1 exposure was lower than 71 μg/kg. On the contrary, the results of HBsAg and presence of urinary aflatoxins alone were significantly associated with 7.3 and 3.4 fold increases in HCC risk [20].

In the study from Wang et al. (2009), there existed a statistical significance in dose-respond relation between AFB1 urinary metabolite and HCC risk, however was not able to demonstrate any persistent synergistic interaction between HBV infection and Aflatoxin, among the cases with HCC diagnosis [22]. In another cohort study it was indicated that AFB-N7-Gua adducts was the mostly detected biomarker in urine to attain HCC risk. It was depicted that aflatoxin was significant agent together with Hepatitis B infection in HCC risk, although specific role of aflatoxin in human liver cancer was not clarified. As distinct from previous endeavors, author put attention on urinary metabolites and DNA-adducts, thus acquired information about only recent exposure to aflatoxins. Assessment of serum albumin aflatoxin adducts might reflect dietary exposure in longer term [23].

Groopman et al. (1993), in China, detected total aflatoxin metabolites in urine with a monoclonal antibody-based radio-immunoassay. Aflatoxin N7-guanine (AFB-N7-Gua), in urine and aflatoxin dietary exposure, was compared extensively in the study. However, pending to methodology- total metabolites were not appropriate for dosimeter measurement for exposure status; eventually paving way to the methodology with HPLC analysis. Linear regression analyses for the urinary levels of biomarker were compared to dietary intake of aflatoxin. Positive correlation was observed between AFB-N7-guanine adduct excretion in urine and toxin intake. Another experiment from the same researchers in Gambia, West Africa, had the results, presenting similarity to the experience in China. As described above, strong evidence for the utility of AFB-N7-guanine in urine as an appropriate molecular dosimetry marker of exposure was proved. In the research, authors also had opportunity to investigate the role of chronic hepatitis B virus infection in the metabolism of aflatoxins. The statistical analysis of the association of hepatitis virus status and the excretion of the AFB-N7- guanine adduct in urine for the utter subjects revealed that AFB-N7-guanine was not related to HBV carrier status. The same study group additionally studied on testing animals (lab-rats) to determine biomarkers for the risk of chronic exposure, rendering a conclusion, that the amount of AFB-N7-guanine in urine represented only 1% of the total aflatoxin metabolites in urine [24].

## Evidence of the uncertain relation between of AFB1 dietary intake and HCC risk

In the report by World Health Organization several studies were evaluated. The data showed that different measures and mechanisms were employed on carcinogenesis than those of employed on codon 249 mutations. It was deduced that; 249 codon mutation of p53 gene mutation might occur if only aflatoxin exposure and codon 249 mutations were introduced in earlier stages of hepatocarcinogenesis [11]. For the verification of the claim, the existence of mutations was analysed through animal testing. In the article, Fuyimoto et al. (1992), AFB1 effects on arginine to serine substation in codon 249 of p53 gene, were observed by analysing nine tumour kinds, induced by AFB1 in nonhuman primates. It was discovered that, in addition to the regions with AFB1 exposure, there were 4 different important hot spots that could be affected by toxin. The mutation was identified in one of the subjects with hepatocellular carcinoma at the second position of codon 175. It was further suggested that p53 mutations were not vital for AFB1 induced carcinogenesis in the subject-animals; thus generating two apparent explanations for these findings: First, the mutation might not occur in non-human primates because of their difference in gene structure than human, and in the absence of HBV- induced chronic hepatitis. Therefore, it was possible that both AFB1 exposure and HBV-induced chronic active hepatitis were necessary for induction of the mutation. Second argument was that the environmental factors may be responsible for the mutations for HCC in human [25].

In one of the epidemiological studies in 1970`s aflatoxin intake was demonstrated as HCC risk factor. However, the same study was lacked the data on liver cancer where the only available data was belonging to the dates between 1945-1950`s. In fact data might show reliability if registered in 1970`s [15]. Another study similar to the similar epidemiological basis, mentioned some disadvantages of methodology; in Transkei region, the samples were collected during 1976 and 1977. Also the number of patients with HCC and the number of food samples, taken in this region were not sufficient for analysis, assessing the individual districts [16]. Although, the study of non- associations Campbell et al. (1990), compromise the contradictory findings; design of the ecological studies were inconsistent with the results. But, in the co-evaluating studies; food sampling methods were deemed insufficient for estimating the extent and exposure of total AFB1. Due to the procedure based on sampling; the particular nutrition products in the markets or in domestic environments; the case of seasonal variation, capable of affecting the levels of contamination, was excluded. In addition, no extensive and individual information about contamination levels were available; eventually yielding to the conclusion that, toxin exposure level was uncertain [8, 15-17]. The case–control study, Baumgarther et al. (2004), found strong association between aflatoxin concentrations in home grown maize, serum B1–albumin adducts, hepatitis B surface antigen titres, and aflatoxicosis status. However, study was restricted to the retrospective setting. The primary case; it was possible that case patients might mislead the researchers about the amount, source, and quality of maize that was consumed differently than controls did. Secondly, the aflatoxin concentrations were measured in sampled maize might had shown difference from those consumed by case patients before the aflatoxicosis emerged. In the study any noticeable association wasn’t found between the number of maize portions, consumed and case status, thwarted by the limited accuracy of the food questionnaires. In addition, it was possible that some case patients might have developed jaundice as a result of undiagnosed medical conditions, unrelated to aflatoxicosis [8]. In the study in Southern Guangxi, different results than the previous studies were presented; yet shown issues concerning the nature of the case-control design and the limited sample size? The authors also mentioned that the evaluation and confirmation would be valuable in cohort studies with a larger sample size [18].

Cohort studies were more accurate to provide valid approach to evaluate the role of AFB1, in contrast to case- control and epidemiological studies with different methods. The methodology in these studies had the obvious and resolute format to determine a true relationship between an exposure and disease outcome [26]. In the study of Quien et al (1994); controls were successfully matched to the cases, as both were recruited and sampled at the same instant and health status. Moreover, it was necessary to enrol large numbers of subjects to ensure accuracy of the case for an appropriate rate. Also only information about non-associative dietary exposure and HCC risk among cohort subjects were demonstrated. Questionnaire data was used to assess dietary intake and market-survey to determine toxin levels in food products within the region. The researchers assigned a quantitative aflatoxin exposure level to each study subject and connected the variable to urinary aflatoxin biomarker status and to a liver cancer risk, and failed to find dose-respond relation with each of the attained parameters. The conclusion was contradictory to another case with well-operated observation of relation between AFB1-N7-Gua biomarker and the cancer risk. In the study; the rate of urinary biomarker detection from single void urine sample was insufficient. Dietary exposure levels were determined through interviews; and, the data reflects the daily variation in urinary aflatoxin levels of a given individual. Also; due to semi quantitative interview instrument, misclassification of diet assessment was quite possible. In the study of Ronald K et al. (1992), it was found out those urinary levels of aflatoxin accurately reflected intake levels of the past 24 hours. The data showed that aflatoxin biomarker rendered more-likely information than the determination of aflatoxin-liver cancer relation by means of dietary assessment [20, 23].

Groopman et al. (1993) also proved that AFB1-N7-Gua was the most convenient biomarker for detection of aflatoxin exposure. The author stressed that the DNA damage by aflatoxin was the first of many other potential factors, involved in the etiopathogenesis of liver cancer. For the convenience; recurrent cytotoxicity, cell proliferation, and nutritional status could distinctively influence the disease process; therefore, author emphasized that the molecular epidemiological investigations, examining only AFB1-N7-Gua, might distort the estimation the cancer risk for an individual. The data, was acquired from the experiment, indicated that the synergy between any biomarker and tumour outcome requires tedious interpretation. In the experiment, effects of HBV among aflatoxin metabolism steps weren’t observed. The author underlined that depending on their results, hepatitis B virus had uncertain path for carcinoma; and, interaction with aflatoxin was unknown. Therefore, according to the author; the proper determination the role of aflatoxin in carcinogenesis was challenging [24].

## Evaluation of the further necessities to determinate the certain role of AFB1 and HBV on risk of HCC

Many ecological studies were suggested a significant role for the AFB1 in HCC [15, 16]. It was indicated in the review study that reducing dietary AFB1 levels to below detectable limits may reduce the prevelance of HCC [27]. Albeit, the etiological role of AFB1 exposure on HCC has been investigated for 50 years, data on the interaction of HBV infection is limited. Hepatitis B virus is an important risk factor for primary liver cancer and presence of this viral infection complicates many of the studies. Interaction between short term AFB1 exposure (dose-dependent) and HBV on P53 gene in HepaRG cells was investigated in the recent study of Lereau et al. (2012). It was observed that as a result of AFB1 treatment HBV proteins (HBsAg, HBeAg and L protein) decreased. AFB1 was assessed as a natural antiviral agent. However, it was mentioned that correlation between the applied doses to the cells in the study and AFB1 liver concentrations after human dietary exposure was not clear. Also, amount of AFB1-N7-guanine between HBV infected and non-infected cells were not different from each other. It was suggested that DNA damage by AFB1 wasn’t affected by HBV infection. The authors emphasized that for the codon 249 of p53 gene precise evaluation of adduct formation levels and DNA repair at base position of relevance HCC, is necessary for further analyses [28].

It was observed in several studies that development of biomarkers for measuring AFB1 metabolites and AFB1-DNA and albumin adducts were resulted in more accurate and reliable than quantification of dietary AFB1 exposure. The results from these studies weren’t totally consistent, and especially in cohort studies quantification of lifetime individual exposure to aflatoxin was extremely difficult. A significant finding of all of these studies was the dose-dependent relationship between aflatoxin exposure and the excretion of the major DNA adduct, AFB-N7-guanine, in urine. As a result rapidly excreted AFB-N7-guanine adducts only reflects relatively recent exposures to AFB1. Temporal relationship between DNA damage and long-term risk from exposure in humans remained to be established [29]. The authors underlined the necessity of the further researches to understand interaction between AFB1 and HBV in regions where HCC cases were common and to observe p53 codon 249 mutations on larger populations [7, 10].

In developing countries with high rate of aflatoxin food contamination, AFB1 research programs are lack of adequate resources in terms of qualified personnel, capital investment, and analytical and technical facilities. In additional, together with all these factors funding support is necessary for comprehensive examinations in these regions. Considering, the reality; great part of the society is suffering from scarcity, thus, consumption of contaminated food commodities is inevitable. Hence for, vaccination programs against hepatitis B virus were recommended as a more realistic and cost-effective strategy for lowering liver cancer incidence than removing aflatoxin from the diet [29, 30].

AFB1 exposure and HBV infection grants pivotal risks for HCC, through two renowned factors, endangering the sanitary status of the public health, in developing states;. Hence forth, the clinical studies, waged to contemplate the issue, are surmounted to bring deeper understanding of the agents, acting on pathogenesis of liver cancer, through set of analysis. The dire need for both point-of-care and clinical approach to annihilate the precursors for HCC exists for resource-low settings and regions. Including all the studies above, incomplete and unclear observations and explanations were done in the regions where public health is widely under threat of AFB1 exposure and HBV. The significant amount of methodological optimizations and brand-new approaches are required for the prospective aspects of the research, sustaining apt opportunity to depict the potent effect of the AFB1. The primary issue is that, the many studies were diverted to endemic regions, demonstrating high HBV rates; and furthermore, the codon 249 mutation of p53 gene wasn’t examined to show any relevance with the AFB1 exposure. The secondary issue is that, the reliability of the HCC clinical data and the prevalence of HBV were limited. Third and the last issue concerns the determination of AFB1 consumption for each individual patient was not convenient, and restrains with only certain food types.

The hurdles, encountered through determination process, plus the lack of single-patient information, also cripples the ecological studies. One must notify that the high relevancy, deduced between variables on an aggregating level didn’t dictate that the similar status might be applied on an individual-level. Nominate, in cohort studies, utilizing the detection of bio-markers from blood/urine samples from patient history – long before the eventual diagnosis – grants a proper representation of the issue, yet failed to deliver the any-possible interaction between hepatitis B and AFB1, in depth. In addition; mutagenic and coexistent effects of AFB1 on hepatitis B weren’t brought to attention, were dismissed without a suggestive cause-&-effect, and were recognized only through evidence-estimation basis.

Solemn reliance on bio-marker levels to construct an abstract sense between two agents was relatively accurate, yet insufficient. Specially; the mutation, was thought to be stemmed from AFB1, occurring on the codon 249 of p53 gene, might be the effective on the carcinogenic factors, such as HBV. The cohort studies were motivated to examine the frequency of the toxin level in the target region of the body. In conclusion; it’s imperative that as long as the cases, suggesting the relation between the mutagenic effect of hepatitis B and the AFB1’s influence on codon 249 mutations, are validated; the scientifically accurate estimations and claims should be constructed. The methods, enforcing the determination of toxin (AFB1) biomarkers, should present accuracy and reliability, contrary to estimation; since previously mentioned adducts quantitate and scrutinize the biologically-effective dose of toxins on HCC for the individual patient.
